# Advances in drug development for hepatocellular carcinoma: clinical trials and potential therapeutic targets

**DOI:** 10.1186/s13046-021-01968-w

**Published:** 2021-05-18

**Authors:** Xiang-Yuan Luo, Kong-Ming Wu, Xing-Xing He

**Affiliations:** 1 Institute of Liver and Gastrointestinal Diseases, Tongji Hospital, Tongji Medical College, Huazhong University of Science and Technology, Wuhan, 430030 China; 2 Hubei Key Laboratory of Hepato-Pancreato-Biliary Diseases, Tongji Hospital, Tongji Medical College, Huazhong University of Science and Technology, Wuhan, 430030 China; 3 Department of Medical Oncology, The Affiliated Cancer Hospital of Zhengzhou University & Henan Cancer Hospital, Zhengzhou, 450008 China; 4 Department of Oncology, Tongji Hospital of Tongji Medical College, Huazhong University of Science and Technology, Wuhan, 430030 China

**Keywords:** Liver cancer, Treatment, Molecular mechanism, Traditional Chinese medicine, Immunotherapy

## Abstract

Although hepatocellular carcinoma (HCC) is one of the deadliest health burdens worldwide, few drugs are available for its clinical treatment. However, in recent years, major breakthroughs have been made in the development of new drugs due to intensive fundamental research and numerous clinical trials in HCC. Traditional systemic therapy schemes and emerging immunotherapy strategies have both advanced. Between 2017 and 2020, the United States Food and Drug Administration (FDA) approved a variety of drugs for the treatment of HCC, including multikinase inhibitors (regorafenib, lenvatinib, cabozantinib, and ramucirumab), immune checkpoint inhibitors (nivolumab and pembrolizumab), and bevacizumab combined with atezolizumab. Currently, there are more than 1000 ongoing clinical trials involving HCC, which represents a vibrant atmosphere in the HCC drug research and development field. Additionally, traditional Chinese medicine approaches are being gradually optimized. This review summarizes FDA-approved agents for HCC, elucidates promising agents evaluated in clinical phase I/II/III trials and identifies emerging targets for HCC treatment. In addition, we introduce the development of HCC drugs in China. Finally, we discuss potential problems in HCC drug therapy and possible future solutions and indicate future directions for the development of drugs for HCC treatment.

**Supplementary Information:**

The online version contains supplementary material available at 10.1186/s13046-021-01968-w.

## Background

Hepatocellular carcinoma (HCC) is one of the deadliest health burdens worldwide [1]. Most patients with HCC have dismal outcomes because of insufficient early diagnosis and few available treatment options for patients with advanced-stage HCC [2].

**Fig. 1:**
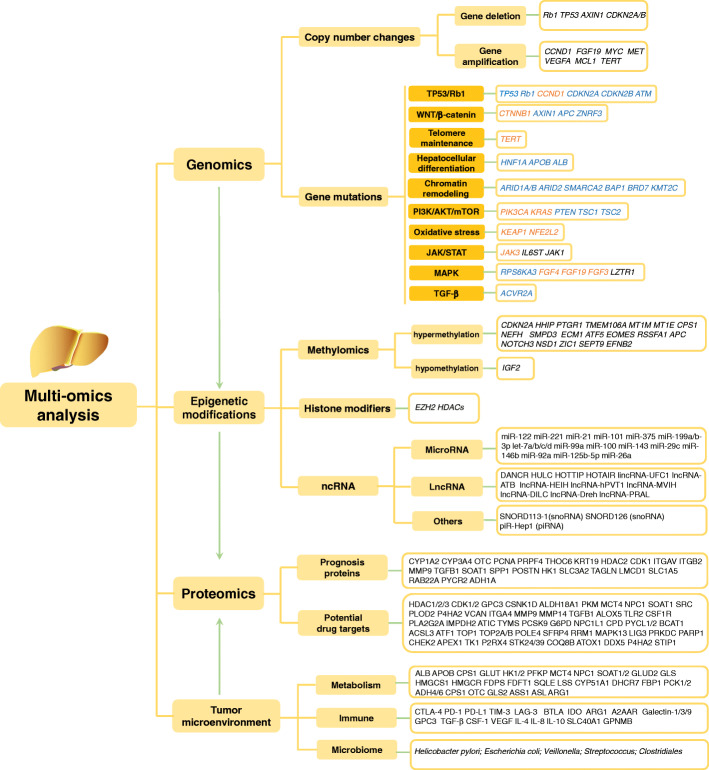
Comprehensive and integrated multiomics analysis of hepatocellular carcinoma (HCC). The main signaling pathways and molecular events in HCC revealed by multiomics are shown. Each mutated gene is marked with an orange font for activation, a blue font for inactivation, and a black font for unclassified

**Fig. 2:**
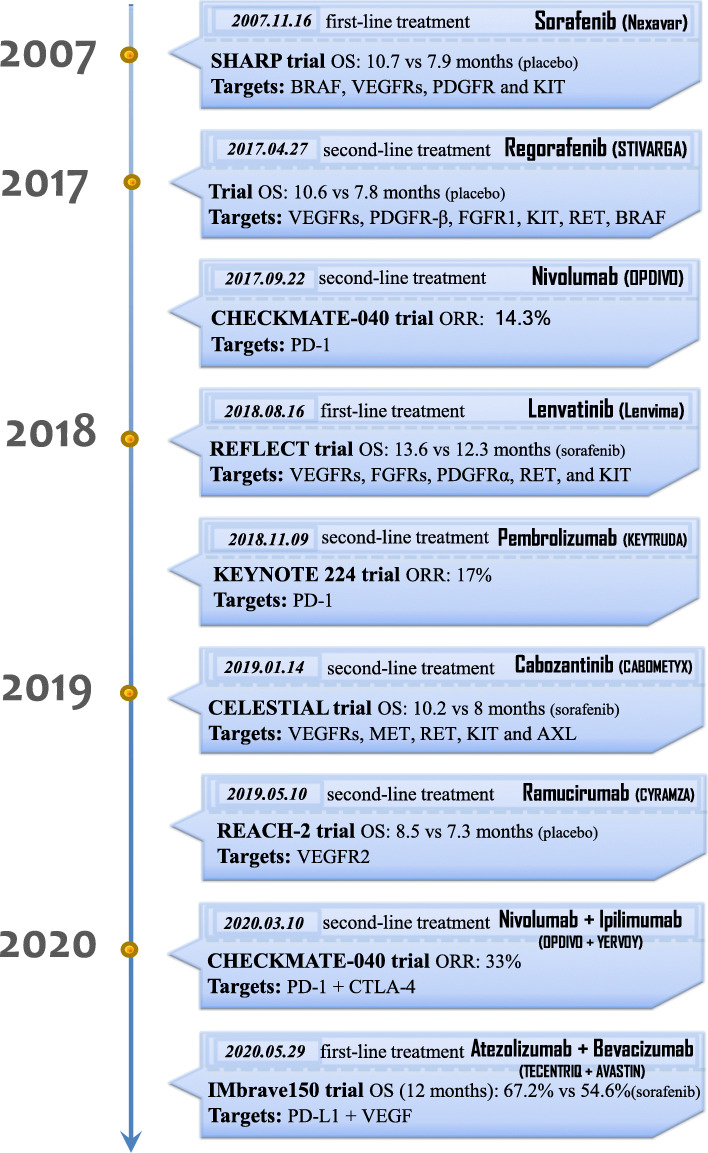
The timeline of FDA-approved drugs for hepatocellular carcinoma (HCC). *Abbreviations:* OS, overall survival; ORR, objective response rate; VEGFR, vascular endothelial growth factor receptor; PDGFR, platelet-derived growth factor receptor; FGFR, fibroblast growth factor receptor; PD-1, programmed cell death-1; PD-L1, programmed cell death ligand 1; CTLA-4, cytotoxic T lymphocyte-associated antigen-4

In recent years, with the rapid advancement of molecular biology techniques, such as high-throughput sequencing, microarrays, and various omics techniques, a more global and thorough understanding of the molecular mechanisms of HCC has been acquired. In particular, the role of epigenetics has been well established and is as important as genetics. The use of integrated multiomics analyses has recently led to outstanding advancements in the in-depth understanding of the molecular hallmarks involved in the initiation and progression of HCC and helped to comprehensively map key signaling pathways and aberrant molecular events in HCC [3–11] (Fig. 1). These valuable technological advances have brought several significant breakthroughs, led to many clinical trials (Table 1), and promoted the approval of multiple drugs by the United States Food and Drug Administration (FDA) (Fig. 2). In 2017–2019, the FDA approved the multikinase inhibitors regorafenib, lenvatinib, cabozantinib, and ramucirumab for HCC [25–28]. Notably, the approval of lenvatinib and of bevacizumab in combination with atezolizumab as two first-line treatment strategies have substantially changed treatment options for HCC as, previously, sorafenib was the only feasible first-line treatment for advanced HCC. Meanwhile, immune checkpoint blockade (ICB) therapy that targets programmed cell death-1 and its ligand (PD-1/PD-L1) and cytotoxic T-lymphocyte-associated protein 4 (CTLA-4) has been remarkably successful for the treatment of melanoma and non-small cell lung cancer, which paved the way for immunotherapy for HCC [29, 30]. In 2017–2018, the FDA accelerated the approval of the anti-PD-1 antibodies nivolumab and pembrolizumab as second-line treatments for patients with HCC [31, 32]. In addition, the technology for extracting active substances from therapies used in traditional Chinese medicine (TCM) has progressed dramatically, facilitating the exploration of the pharmacological mechanisms underlying TCM. All these developments show optimistic prospects for HCC drug treatment.

**Table 1:** Agents with significant effects on hepatocellular carcinoma (HCC) in clinical trials

Agent	Targets	Study design	Sample size	OS (months)	Efficacy	Safety
Sorafenib vs. Placebo (SHARP) [12]	VEGFRs, KIT, PDGFRs, and RAF	Phase III; First-line; Randomised; Multicenter; Double-blind	*n* = 602	Srafenib: 10.7 Placebo: 7.9 HR: 0.69 (*P* < 0.001)	**TTRP (months)**: Srafenib: 5.5; Placebo: 2.8 HR: 0.58 (*P* < 0.001) **ORR**: 2% **DCR**: 43%	TRAEs: 80%; SAEs: 52%;
Nivolumab (CheckMate 040) [13]	PD-1	Phase I/II; Second-line; Multicentre; Open-label; Non-comparative	DES (*n* = 48) DEX (*n* = 214)	15 (DES)	**DES**:TTP: 3.4 months ORR: 15% DCR: 58% **DEX**:TTP: 4.1 months ORR: 20% DCR: 64%	**DES:**Grade 3/4 AEs: 25%; SAEs: 6% **DEX:**Grade 3/4 AEs: 19%; SAEs: 4%
Pembrolizumab (KEYNOTE 224) [14]	PD-1	Phase II; Second-line; Non-randomised; Multicentre; Open-label	*n* = 104	12.9	**TTP**: 4.9 months **PFS**: 4.9 months **ORR**: 17% **DCR**: 62%	TRAEs: 73%; Grade 3/4 AEs: 25%; SAEs:15%
Tremelimumab [15]	CTLA-4	Phase II; Non-controlled; Multicentre; Open-label	*n* = 21	8.2	**TTP**: 6.48 months **PRR**: 17.6% **DCR**: 76.4%	TRAEs: Skin rash, Fatigue, Diarrhea
Lenvatinib vs Sorafenib (REFLECT) [16]	VEGFR1–3, FGFR1–4, PDGFRα, RET, and KIT	Phase III; First-line; Multicentre; Non-inferiority; Open-label	*n* = 954	Lenvatinib:13.6 Sorafenib: 12.3 HR: 0.92	**TTP (months)**: Lenvatinib: 8.9; Sorafenib: 3.7 HR: 0.63 (*P* < 0.0001) **PFS (months):** Lenvatinib: 7.4; Sorafenib: 3.7 HR: 0.66 (*P* < 0.0001) **DCR**: 75.5% **ORR**: 24.1%	TEAEs: 99%; Grade ≥ 3 TEAEs: 75%; STEAEs: 43%
(Chinese subgroup) [17]				Lenvatinib:15 Sorafenib: 10.2 HR: 0.73 (*P* = 0.026)	**PFS (months):** Lenvatinib: 9.2; Sorafenib: 3.6 HR: 0.55 (*P* = 0.00001)	
Regorafenib vs Placebo (RESORCE) [18]	VEGFR1–3, PDGFR-β, FGFR1, KIT, RET and B-RAF	Phase III; Second-line; Randomised; International; Double-blind	*n* = 573	Regorafenib: 10.6 Placebo: 7.8 HR: 0.63 (*P* < 0.0001)	**TTP (months):** Regorafenib: 3.2; Placebo: 1.5 HR: 0.44 (*P* < 0.0001) **PFS (months)**: Regorafenib: 3.1; Placebo: 1.5 HR: 0.46 (*P* < 0.0001) **DCR**: 65% **ORR**: 11%	TEAEs: 100%; Grade 3/4 TEAEs: 67%; SAEs: 44%
Cabozantinib vs Placebo [19]	VEGFR1–3, MET, RET, KIT and AXL	Phase III; Second-line; Randomised; Double-blind	*n* = 707	Cabozantinib: 10.2 Placebo:8.0 HR:0.76 (*P* = 0.005).	**PFS (months)**: Cabozantinib: 5.2; Placebo: 1.9 HR: 0.44 (*P* < 0.001) **DCR**: 64% **ORR**: 4%;	AEs: 99%; Grade 3/4 AEs: 68%; SAEs: 50%
Ramucirumab vs Placebo (REACH-2) [20]	VEGFR2	Phase III; Second-line; Randomised; Double-blind (AFP ≥400 ng/mL)	*n* = 292	Ramucirumab: 8.5 Placebo: 7.3 HR: 0.710 (*P* = 0.0199)	**PFS (months):** Ramucirumab: 2.8; Placebo: 1.6 HR: 0.452 (*P* < 0.0001) **DCR**: 59.9% **ORR**: 4.6%	Grade ≥ 3 AEs: ≥5%
Apatinib [21]	VEGFR2	Phase II; First-line; Randomised; Multicentre; Open-label; Dose-finding	*n* = 121	9.7 (850 mg qd) 9.8 (750 mg qd)	**TTP (months):** 4.2 (850 mg qd); 3.3 (750 mg qd) **DCR:** 48.57% (850 mg qd); 37.25% (750 mg qd)	AEs: 2%
Bevacizumab+Atezolizumab vs Sorafenib (IMbrave150) [22]	VEGF+PD-L1	Phase III; First-line; Randomised; Multicentre; Open-label	*n* = 501	B + A:67.2% Sorafenib:54.6 (12 months)	**PFS (months):** B + A: 6.8; Sorafenib: 4.3 **DCR**:73.6% **ORR**:27.3%	AEs: 98.2%; Grade 3/4 AEs: 56.5%
Lenvatinib+Pembrolizumab [23]	VEGFR1–3, FGFR1–4, PDGFRα, RET, and KIT+PD1	Phase Ib; First-line; Multicentre; Open-label	*n* = 104	22.0	**TTP (months):** 9.7 **PFS (months):** 8.6–9.3 **ORR**: 36–46%	AEs: 99%; Grade ≥ 3 TRAEs: 67%; SAEs: 65%
Nivolumab+Ipilimumab (CheckMate 040) [24]	PD-1+ CTLA-4	Phase I/II; Second-line; Multicentre; Open-label	*n* = 148	arm A*: 22.8 arm B*: 12.5 arm C*: 12.7	**ORR**: 32%(A); 27%(B); 29%(C) **DOR (months)**: no reached(A); 15.2(B); 21.7(C)	TRAEs: 94%(A); 71%(B); 79%(C)

*Abbreviations*: *VEGFRs* vascular endothelial growth factor receptors, *PDGFRs* platelet-derived growth factor receptors, *PD-1* programmed cell death-1, *CTLA-4*, cytotoxic T lymphocyte-associated antigen-4, *FGFR1–4* fibroblast growth factor receptor 1–4, *OS* overall survival, *HR* hazard ratio, *TTP* time to progress, *TTRP* Time to radiologic progression, *ORR* objective response rate, *DCR* disease control rate, *PFS* progress free survival, *AEs* adverse events, *TRAEs* treatment-related AEs, *SAEs* serious AEs, *TEAEs* treatment-emergent AEs, *STEAEs* serious treatment-emergent AEs, *DES* dose-escalation, *DEX* dose-expansion, *DOR* duration of response

*arm A: Give 1 mg/kg of nivolumab and 3 mg/kg of ipilimumab every 3 weeks (4 doses), then 240 mg of nivolumab every 2 weeks

*arm B: Give 3 mg/kg of nivolumab and 1 mg/kg of ipilimumab every 3 weeks (4 doses), then 240 mg of nivolumab every 2 weeks

*arm C: Give 3 mg/kg of nivolumab every 2 weeks and 1 mg/kg of ipilimumab every 6 weeks

In this review, we summarize the FDA-approved agents for HCC, clarify the promising agents being evaluated in phase I/II/III trials as reported at ClinicalTrials.gov (supported by the US National Library of Medicine) from the molecular mechanism perspective, and outline the emerging targets for HCC treatment. We introduce the development of HCC drugs in China. In addition, we discuss the potential problems in HCC drug treatment discovered in recent years and present some feasible solutions. Finally, we indicate the possible future directions of drug development for HCC treatment.

## Agents approved for HCC

### First-line treatment

#### Sorafenib

Sorafenib is a multikinase inhibitor that blocks the activity of *RAF-1*, *BRAF*, *VEGFRs*, *PDGFR,* and *KIT* receptors involved in cell proliferation and angiogenesis [33, 34]. It has been the standard first-line treatment for patients with advanced HCC since the FDA approved sorafenib for HCC in 2007 [35]. The Sorafenib Hepatocellular Carcinoma Assessment Randomized Protocol (SHARP) trial and the sorafenib Asia-Pacific (AP) trial have previously demonstrated the benefit of sorafenib compared with a placebo for patients with advanced HCC without systemic treatment [12, 36]. Herein, the median overall survival (OS) of the sorafenib group was prolonged by approximately 2–3 months, and the secondary endpoints were also significantly favorable in both trials [12, 36]. However, the partial response rate (PRR) of the sorafenib group was relatively low (2% in SHARP and 3.3% in AP), and the participants did not achieve a complete response in either trial [12, 36]. In addition, the clinical application of sorafenib is limited by tumor heterogeneity, tumor escape, and the lack of predictive biomarkers for response to the treatment [37, 38]. Regarding the safety profile, the most frequent grade 3/4 sorafenib-related adverse events (AEs) are hand-foot skin reaction (HFSR), fatigue, and diarrhea [12, 36] (Table 1).

Because of patients’ inadequate response to sorafenib, its management is critical to improve the efficacy, especially to manage AEs and select patients most likely to respond [39]. HFSR (the most common AE) is the most noteworthy challenge. Although various methods are used to prevent or minimize the effect of HFSR, including urea-based creams and the dose-reduction of sorafenib, clinical monitoring is necessary for the first 2 months during sorafenib therapy owing to the current high incidence of HFSR [39, 40]. Importantly, some AEs, such as skin-related AEs, may serve as potential biomarkers to predict sorafenib efficacy due to the significant correlation between AEs and survival [41, 42]. For the management of patient selection, the aim is to identify the patients most likely to respond to sorafenib treatment. Bruix et al. [43] thoroughly analyzed the results of two phase III trials, which showed that all patient subgroups treated with sorafenib had a survival benefit. Nevertheless, for patients with HCV, liver-confined disease (without EHS), or a lower neutrophil: lymphocyte ratio, sorafenib has a greater benefit [43].

#### Lenvatinib

Lenvatinib, which targets *VEGFR 1–3*, *FGFR 1–4*, *PDGFR α*, *RET*, and *KIT*, is the first-line systemic therapy drug for advanced HCC [17]. In 2018, the results of a randomized phase III trial (REFLECT) demonstrated that lenvatinib was noninferior to sorafenib in terms of OS [16]. The median OS was 13.6 months for lenvatinib compared to 12.3 months for sorafenib [16]. And all secondary endpoints (progression-free survival <PFS>, time to progression <TTP>, and objective response rate < ORR>) exhibited significant improvement with lenvatinib compared with sorafenib [16] (Table 1). The common AEs included hypertension (42%), diarrhea (39%), decreased appetite (34%), and decreased weight (31%) [16]. In addition, since hepatic arterial infusion chemotherapy (HAIC) is mainly applied for localized advanced HCC and has shown more beneficial long-term outcomes in Japan [44], the efficacy and safety of lenvatinib combined with HAIC for patients with advanced HCC is being evaluated in a randomized controlled phase III trial (NCT03775395).

#### Bevacizumab plus Atezolizumab

In recent years, immunotherapy, especially the ICB strategy, has been frequently explored for tumor treatment and has shown substantial clinical efficacy. ICB is a way to directly protect against immune checkpoint proteins. Once these proteins bind to specific ligands in the tumor environment, they suppress immune cell immune function, thereby blocking the antitumor immune response [45]. PD-L1 is one of the ligands of the immune checkpoint protein PD-1.

In 2020, the FDA approved the combination strategy of bevacizumab (anti-VEGF antibody) plus atezolizumab (anti-PD-L1 antibody) as a first-line treatment for unresectable HCC based on the results of the IMbrave150 trial (NCT03434379) [22, 46]. In this phase III trial, the efficacy of bevacizumab combined with atezolizumab was compared with sorafenib in 501 patients with HCC without prior systemic therapy [22]. The combination treatment resulted in a substantially better outcome than sorafenib monotherapy, increasing the OS by 12.6% at 12 months and markedly prolonging the PFS by 2.5 months [22] (Table 1). In addition, there was no significant difference in the incidence of AEs between the combination treatment group and sorafenib group, and no AEs beyond the safety profile of the single drug and the effects of the underlying disease were found [22]. Therefore, bevacizumab in combination with atezolizumab treatment was determined to be relatively safe for patients with HCC.

### Second-line treatment

#### Regorafenib

In April 2017, regorafenib became the first agent approved by the FDA as a second-line therapy for patients with advanced HCC who progressed after sorafenib treatment [25]. Regorafenib is a multikinase inhibitor that mainly targets angiogenic factors, including *VEGFR1–3*, *PDGFR-β*, *FGFR1*, *KIT*, *RET,* and *BRAF*. Regorafenib possesses even more robust inhibitory activity than sorafenib [47]. Based on the results of a randomized, placebo-controlled phase III trial in 2017 (NCT01774344), regorafenib was more potent than the placebo with a mortality reduction of 37% (median survival of 10.6 months for regorafenib versus 7.8 months for placebo) [18, 48] (Table 1). Of note, this trial only enrolled patients with Child-Pugh A liver function to decrease the impact of deteriorative liver function on the trial outcomes [18]. Furthermore, biomarker-related studies regarding this treatment approach have still not progressed.

#### Cabozantinib

Cabozantinib is a multitarget tyrosine kinase inhibitor (TKI) that mainly blocks *VEGFR2* and *MET*, although it has effects on *VEGFR1/3*, *RET*, *KIT,* and *AXL* [49]. Its approval was based on a randomized, placebo-controlled phase III trial (CELESTIAL) that showed that the median survival was prolonged by 2.2 months in the cabozantinib group (NCT01908426) [19] (Table 1). In particular, among patients who previously received sorafenib alone, the median survival in the cabozantinib group was 11.3 months [12, 19]. This finding indicated the advantage of cabozantinib as a second-line treatment for improving patient survival compared with sorafenib alone (median survival of 10.7 months) [12, 19]. Additionally, cabozantinib has the potential to partially resolve the problem of MET-induced sorafenib resistance, which has been shown in previously reported studies [50, 51]. Furthermore, there is evidence that *MET*, *HGF*, *GAS6*, *VEGF-A*, *ANG2*, and *IL-8* serve as biomarkers to predict the prognosis of patients treated with cabozantinib [52].

#### Ramucirumab

Ramucirumab is a recombinant IgG1 monoclonal antibody (mAb) that specifically targets *VEGFR2*. In 2015, Zhu et al. [53] completed a randomized phase III trial evaluating ramucirumab as second-line therapy for patients with advanced HCC (REACH). Unfortunately, they failed to obtain the anticipated outcome [53]. Interestingly, in contrast to the fact that a high level of α-fetoprotein (AFP) suggests a poor prognosis, they found that an elevated baseline level of AFP (≥400 ng/mL) contributed to prolonged survival with ramucirumab treatment [53]. Hence, a subsequent REACH-2 trial re-evaluated ramucirumab with a new inclusion criterion of AFP ≥ 400 ng/mL (NCT02435433). According to a report from the American Society of Clinical Oncology (ASCO) annual meeting in June 2018, the efficacy of ramucirumab was improved (median OS of 8.5 months for ramucirumab group versus 7.3 months for placebo group), and the safety profile was manageable (Table 1) [20]. This first biomarker-based trial with favorable results in HCC led to the FDA approval of ramucirumab as a second-line treatment for patients with HCC patients AFP level was ≥400 ng/mL [28, 54].

#### Immune checkpoint inhibitors (ICIs)

##### Nivolumab

PD-1 is one of the essential immune checkpoints and is highly expressed in exhausted T cells, B cells, and myeloid cells [55, 56]. In 2017, based on positive results from an open-label, noncomparative, phase I/II dose escalation and expansion trial (CheckMate 040) [13] (NCT01658878), the anti-PD-1 mAb nivolumab resulted in a 15-month OS. Regardless of the therapeutic line for advanced HCC, the ORR with nivolumab was 15–20%, much better than the 2% ORR with sorafenib [13, 35] (Table 1). It is worth noting that there is clear evidence that 82% of nonintervention patients have an ORR of 23% and OS of 9 months, which provides a basis for the application of nivolumab as a first-line treatment for patients with advanced HCC [13]. In 2019, the European Society for Medical Oncology (ESMO) reported clinical trial (CheckMate 459) data that showed that nivolumab paralleled the efficacy of sorafenib as a first-line treatment for advanced HCC, though there was no statistically significant difference in OS (16.4 months median OS for nivolumab versus 14.7 months for sorafenib) [57]. In addition, nivolumab was reported to be safer than sorafenib (22% AEs verse 46% AEs) [57]. Nevertheless, similar to sorafenib, there is no reliable response biomarker for nivolumab. Moreover, it is unclear which groups of patients with advanced HCC benefit the most from nivolumab treatment. All of the above are issues that require more attention in future research.

##### Pembrolizumab

Pembrolizumab is another PD-1 mAb that has been granted accelerated approval by the FDA for HCC treatment. In 2018, the results of a single-arm phase II trial (KEYNOTE 224) showed that pembrolizumab elicited an encouraging response and had manageable toxicity in HCC patients [14] (Table 1). Specifically, the ORR of patients treated with pembrolizumab reached 17%, with one patient achieving complete response and 17 achieving partial responses [14]. The median OS was 12.9 months, and 54% of patients were alive after 12 months [14]. However, this trial had some limitations, namely the lack of a randomized control arm, the absence of an evaluation of the progression of patients treated with sorafenib, and inadequate analysis between biomarkers and pembrolizumab treatment response [14]. Subsequently, two large randomized controlled phase III trials, KEYNOTE-240 (NCT02702401) and KEYNOTE-394 (NCT03062358), were conducted to further test pembrolizumab treatment for HCC. According to the latest report of the KEYNOTE-240 clinical trial, pembrolizumab failed to meet the statistically prespecified OS and PFS [58]. Nevertheless, the OS and PFS in the pembrolizumab group were improved compared to the placebo group (13.9-month OS and 3.0-month PFS with the pembrolizumab; 10.6-month OS and 2.8-month PFS with the placebo) [58]. The ORR was 18.3% in the pembrolizumab group, significantly higher than the 4.4% ORR in the placebo group [58]. In summary, this was the first phase III trial to reveal the efficacy of checkpoint inhibitors for the treatment of HCC and provides evidence to support pembrolizumab’s accelerated approval.

##### Nivolumab plus ipilimumab

The combination of ICIs is a novel and effective treatment strategy for HCC. In 2020, the FDA accelerated the approval of nivolumab plus ipilimumab (anti-CTLA-4 antibody) as a second-line treatment for HCC based on the results of the CheckMate 040 clinical trial [24]. The results of this trial indicated that the ORR of the arm A dosage regimen (nivolumab 1 mg/kg and ipilimumab 3 mg/kg every 3 weeks, then nivolumab 240 mg every 2 weeks) reached 32%, which was higher than that of the other two dosage regimens [24] (Table 1). However, given the limitations of this trial, a randomized controlled trial involving a larger patient sample with stratification will be needed in the future. This combination regimen is currently being evaluated as a first-line treatment for HCC in a phase III trial (NCT04039607). In addition, the CheckMate 040 trial results showed the following clinically meaningful outcomes: the ORR of nivolumab, ipilimumab, and cabozantinib combination treatment for HCC was 26%, the disease control rate (DCR) was 83%, and the median PFS was 6.8 months [59]. The median OS has not yet been reached [59].

## Promising agents in clinical trials for HCC

**Fig. 3:**
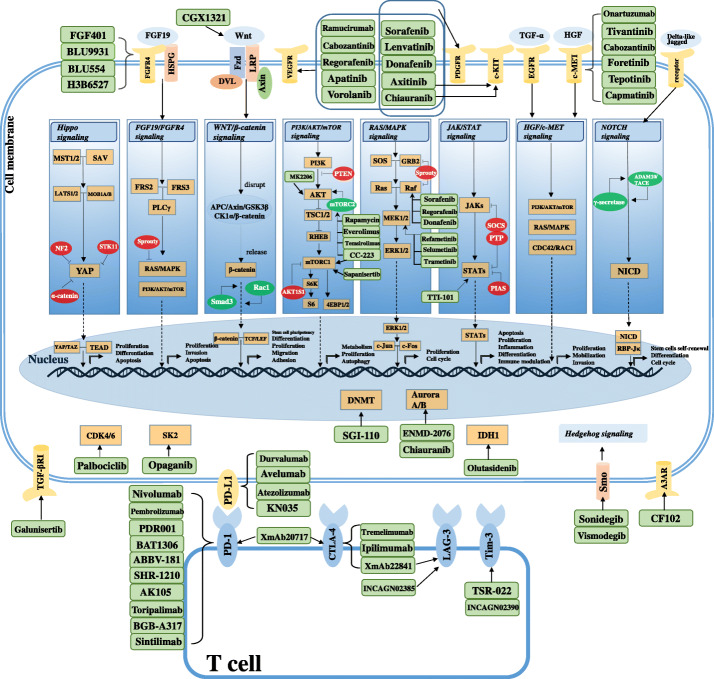
The main signaling pathways and molecular targets of targeted therapy for hepatocellular carcinoma (HCC). We summarize several crucial and well-established signaling pathways that drive HCC initiation and progression, as well as the core functional molecules and their partial regulators in these signaling cascades, and then list their targeted agents currently in clinical studies. *Abbreviations:* MST1/2, macrophage stimulating 1 and 2; SAV, salvador family WW domain containing protein; LATS1/2, large tumor suppressor kinases 1 and 2; MOB1A/B, MOB kinase activators 1A and B; YAP, yes-associated protein 1; NF2, neurofibromin 2; STK11, serine/threonine kinase 11; TEAD, TEA domain transcription factor; WWTR1/TAZ, WW domain containing transcription regulator 1; FGF19, fibroblast growth factor 19; FGFR4, fibroblast growth factor receptor 4; HSPG, heparin sulphate proteoglycans; FRS2/3, fibroblast growth factor receptor substrate 2 and 3; PLCγ, phospholipase C gamma 1; Fzd, frizzled; LRP, low-density lipoprotein receptor-related protein; DVL, dishevelled; APC, adenomatous polyposis coli; GSK3β, glycogen synthase kinase 3β; CK1α, casein kinase 1α; Smad3, mothers against decapentaplegic homolog 3; Rac1, Rac family small GTPase 1; TCF/LEF, T-cell factor/lymphoid enhancer factor; PI3K, phosphoinositide 3-kinase; PTEN, phosphatase and tensin homologue; TSC1/2, tuberous sclerosis 1/2; mTORC1/2, mammalian target of rapamycin complex 1/2; RHEB, RAS homologue enriched in brain; S6K, S6 kinase; AKT1S1, Akt1 substrate 1; 4EBP1/2, eukaryotic translation-initiation factor 4E-binding protein 1/2; MAPK, mitogen-activated protein kinase; SOS, SOS Ras/Rac guanine nucleotide exchange factor; GRB2, growth factor receptor bound-2; MEK1/2, mitogen-activated protein kinase kinase 1/2; ERK1/2, extracellular signal-regulated kinase 1/2; JAKs, janus kinases; STATs, signal transducers and activators of transcription; SOCS, suppressors of cytokine signaling; PTP, protein tyrosine phosphatase; PIAS, protein inhibitors of activated STAT; EGFR, epidermal growth factor receptor; TGF-α, transforming growth factor α; HGF, hepatocyte growth factor; CDC42, cell division cycle 42; ADAM10/TACE, tumor necrosis factor–converting enzyme; NICD, notch intracellular domain; RBP-Jκ, DNA-binding recombination signal-binding protein Jκ; VEGFR, vascular endothelial growth factor receptor; PDGFR, platelet-derived growth factor receptor; TGF-βR1, transforming growth factor beta receptor 1; CDK4/6, cyclin dependent kinase 4/6; SK2, sphingosine kinase 2; DNMT, DNA methyltransferase; IDH1, isocitrate dehydrogenase (NADP(+)) 1; Smo, smoothened, frizzled class receptor; A3AR, adenosine A3 receptor; PD-1, programmed cell death-1; PD-L1, programmed cell death ligand 1; CTLA-4, cytotoxic T lymphocyte–associated antigen-4; LAG-3, lymphocyte activating 3; Tim-3, T-Cell immunoglobulin and mucin domain-containing molecule 3

In previous decades, antiangiogenic therapy has been the core strategy against HCC, since angiogenesis is an indispensable condition for the survival of malignant tumors. With the continuous advancement of science and technology, substantial progress has been made in the study of the genome and epigenome of tumors and has provided a profound and comprehensive understanding of the hepatocarcinogenesis mechanism, thereby promoting the development of more potential HCC treatment drugs involving multiple mechanisms. (shown in Fig. 3). Currently, there are more than 1000 ongoing clinical trials related to HCC, which represents a vibrant atmosphere in the area of drug research for HCC.

### Agents in phase III trials

Based on the excellent antitumor effects of ICIs in clinical trials for HCC, researchers have applied therapeutic strategies combining these ICIs with other agents to improve their efficacy (Table 2), many of which have obtained promising results in early-stage clinical trials and are currently being tested in phase III trials. In addition, there are other promising agents for HCC treatment currently being evaluated in phase III trials.

**Table 2:** Combination treatment of hepatocellular carcinoma (HCC) in clinical trials

Drug	Targets	Stage and conditions	Phase	Primary endpoint(s)	ClinicalTrials.gov Identifier	Study start
**Immunotherapy plus Anti-angiogenesis**						
Atezolizumab plus Lenvatinib or Sorafenib	PD-L1 + VEGFRs, FGFRs, PDGFR α, RET, KIT and RAF	Advanced; Second-line	III	OS	NCT04770896	2021
SHR-1210 plus Apatinib	PD-1 + VEGFR-2	Advanced; First-line	III	OS/PFS	NCT03764293	2019
CS1003 plus Lenvatinib	PD-1 + VEGFRs, FGFRs, PDGFR α, RET and KIT	Advanced; First-line	III	OS/PFS	NCT04194775	2019
Durvalumab plus Bevacizumab	PD-L1 + VEGFA	High risk of recurrence; Second-line	III	RFS	NCT03847428	2019
Atezolizumab plus Bevacizumab	PD-L1 + VEGFA	Locally advanced or metastatic; First-line	III	OS/PFS	NCT03434379	2018
Atezolizumab plus Cabozantinib	PD-L1 + VEGFR, MET, RET, KIT and AXL	Advanced; First-line	III	OS/PFS	NCT03755791	2018
Pembrolizumab plus Lenvatinib	PD-1 + VEGFRs, FGFRs, PDGFR α, RET and KIT	Advanced; First-line	III	OS/PFS	NCT03713593	2018
Nivolumab plus Sorafenib	PD-1 + VEGFRs, KIT, PDGFRs, and RAF	Locally Advanced or Metastatic; First-line	II	MTD/ORR	NCT03439891	2018
Avelumab plus Regorafenib	PD-L1 + VEGFR1–3, PDGFR-β, FGFR1, KIT, RET and B-RAF	Advanced or metastatic	I/II	RP2D/ORR	NCT03475953	2018
Nivolumab plus Cabozantinib	PD-1 + VEGFR, MET, RET, KIT and AXL	Locally Advanced; Neoadjuvant	I	AEs	NCT03299946	2018
Nivolumab plus Bevacizumab	PD-1 + VEGFA	Advanced or Metastatic	I	AEs/MTD or RP2D	NCT03382886	2018
Durvalumab plus Cabozantinib	PD-L1 + VEGFR, MET, RET, KIT and AXL	Advanced; Second-line	I	MTD	NCT03539822	2018
Nivolumab plus Vorolanib	PD-1 + VEGFR, PDGFR	/	I	RP2D	NCT03511222	2018
PDR001 plus Sorafenib	PD-1 + VEGFRs, KIT, PDGFRs, and RAF	Advanced; First-line	I	AEs	NCT02988440	2017
Pembrolizumab plus Regorafenib	PD-1 + VEGFR1–3, PDGFR-β, FGFR1, KIT, RET and B-RAF	Advanced; First-line	I	AEs/DLTs	NCT03347292	2018
Durvalumab plus Ramucirumab	PD-L1 + VEGFR2	Advanced or metastatic	I	DLTs	NCT02572687	2016
**Immunotherapy plus other agents**						
IBI310 plus Sintilimab	CTLA-4 + PD-1	Advanced; First-line	III	OS/ORR	NCT04720716	2021
Nivolumab plus Ipilimumab	PD-1 + CTLA-4	Advanced; First-line	III	OS	NCT04039607	2019
Durvalumab plus Tremelimumab	PD-L1 + CTLA-4	Advanced; First-line	III	OS	NCT03298451	2017
TSR-042 plus TSR-022	PD-1 + TIM-3	Locally advanced or metastatic	II	ORR	NCT03680508	2018
Pembrolizumab plus Bavituximab	PD-1 + PS	Advanced; First-line	II	ORR	NCT03519997	2018
Nivolumab plus BMS-986205	PD-1 + IDO1	Advanced; First-line	I/II	AEs/ORR	NCT03695250	2018
Pembrolizumab plus Epacadostat	PD-1 + IDO1	/	I/II	DLTs/ORR	NCT02178722	2014
Pembrolizumab plus INCAGN01876 plus Epacadostat	PD-1 + GITR+IDO1	Advanced	I/II	AEs/ORR	NCT03277352	2017
Nivolumab plus Galunisertib	PD-1 + TβRI	Advanced; Recurrent	I/II	MTD	NCT02423343	2015
Nivolumab plus Avadomide	PD-1 + CRBN	Unresectable	I/II	DLT/AEs/ORR	NCT02859324	2016
Pembrolizumab plus VSV-IFNβ-NIS	PD-1 + Oncolytic virus	Refractory	I	ORR/AEs	NCT03647163	2019
Durvalumab plus Guadecitabine	PD-L1 + DNMT	Advanced; Metastatic	I	AEs/ORR	NCT03257761	2018
Pembrolizumab plus XL888	PD-1 + Hsp90	Advanced; Metastatic	I	RP2D	NCT03095781	2017
Pembrolizumab plus Vaccine	PD-1 + Modified Vaccinia Virus Ankara Vaccine Expressing p53	Unresectable; Second-line	I	Tolerability	NCT02432963	2015
PDR001 plus NIS793	PD-1 + TGF-β	Advanced	I	DLTs/AEs	NCT02947165	2017
Nivolumab plus SF1126	PD-1 + PI3K	Advanced	I	DLT	NCT03059147	2017
**Other combination**						
Apatinib plus Capecitabine	VEGFR-2 + DNA/RNA Synthesis	Advanced	II	TTP	NCT03114085	2017
Temsirolimus plus Sorafenib	mTOR+VEGFRs, KIT, PDGFRs, and RAF	Advanced; First-line	II	TTP	NCT01687673	2012
Trametinib plus Sorafenib	MEK 1/2 + VEGFRs, KIT, PDGFRs, and RAF	Advanced	I	MTD	NCT02292173	2014
CVM-1118 plus Sorafenib	VM + VEGFRs, KIT, PDGFRs, and RAF	Advanced	II	ORR	NCT03582618	2018
mFOLFOX plus Sorafenib	DNA Synthesis+VEGFRs, KIT, PDGFRs, and RAF	/	II	TTP	NCT01775501	2013
Erlotinib plus Bevacizumab	EGFR+VEGFA	Advanced; Second-line	II	PFS (16 W)	NCT01180959	2011
TRC 105 plus Sorafenib	Endoglin+VEGFRs, KIT, PDGFRs, and RAF	/	I/II	MTD/ORR	NCT02560779	2016
Enzalutamide plus Sorafenib	AR + VEGFRs, KIT, PDGFRs, and RAF	Advanced; First-line	I/II	PFS	NCT02642913	2015
Napabucasin or Amcasertib plus Sorafenib	STAT3, cancer stemness kinase+VEGFRs, KIT, PDGFRs, and RAF	Advanced; First-line	I/II	RP2D/AEs/AA	NCT02279719	2014
ADI-PEG 20 plus FOLFOX	Arginine+DNA Synthesis	Advanced	I/II	ORR	NCT02102022	2014
FATE-NK100 plus Cetuximab or Trastuzumab	NK cell immunotherapy+EGFR or EGFR2	EGFR1+ or HER2+; Advanced	I	DLT	NCT03319459	2018
Navitoclax plus Sorafenib	Bcl-2 + VEGFRs, KIT, PDGFRs, and RAF	Relapsed or refractory	I	MTD/AEs	NCT02143401	2014

*Abbreviations*: *PD-1* programmed cell death-1, *VEGFR* vascular endothelial growth factor receptor, *VEGF* vascular endothelial growth factor, *PD-L1* programmed cell death ligand 1, *FGFR* fibroblast growth factor receptor, *FGF* fibroblast growth factor, *PDGFR* platelet derived growth factor receptor, *CTLA-4* cytotoxic T lymphocyte–associated antigen-4, *TIM-3* T-Cell immunoglobulin and mucin domain-containing molecule 3, *PS* phosphatidylserine, *IDO1* indoleamine 2,3-Dioxygenase 1, *GITR* glucocorticoid-induced tumor necrosis factor receptor, *TβRI* transforming growth factor beta receptor 1, *CRBN* cereblon, *DNMT* DNA methyltransferase, *Hsp90* heat shock protein 90, *TGF-β* transforming growth factor beta, *PI3K* phosphatidylinositol 3-kinase, *mTOR* mechanistic target of rapamycin kinase, *VM* vasculogenic mimicry, *EGFR* epidermal growth factor receptor, *AR* androgen receptor, *STAT3* signal transducer and activator of transcription 3, *OS* overall survival, *PFS* progress free survival, *MTD* maximum tolerated dose, *ORR* objective response rate, *RP2D* recommended phase II dose, *AEs* adverse events, *DLT* dose limited toxicity, *TTP* time to progress, *AA* antitumor activity, *RFS* recurrence free survival

#### ICIs combined with angiogenesis inhibitors

Previous studies have shown that antiangiogenesis and immunotherapy have a synergistic antitumor effect, jointly inducing tumor immune stimulation and vascular remodeling [60–62]. Furthermore, the relatively different AEs that arise with angiogenesis inhibitor and ICI treatments may facilitate their use as a combination therapy [63]. Specifically, the combination of lenvatinib and PD-1 mAb enhances antitumor capacity by minimizing monocytes and macrophages (antitumor immunity inhibition) to increase the activation of CD8+ T cells [64]. Early-stage clinical trials have shown promising outcomes with lenvatinib combined with pembrolizumab as the first-line treatment for unresectable HCC [23]. In this combination therapy group, the ORR was 36–46%, PFS was 8.6–9.3 months, OS was 22 months, and the toxicity was manageable (NCT03006926) [23] (Table 1). This combination therapy was granted breakthrough therapy designation by the FDA in 2020 for use as a first-line treatment [23]. Currently, a phase III trial evaluating lenvatinib plus pembrolizumab as the first-line treatment of unresectable HCC is underway (NCT03713593). In addition, Xu et al. [65] conducted a phase I/II trial of apatinib (anti-VEGFR2) plus SHR-1210 (anti-PD-1 mAb) for HCC and gastric cancer in 2018. In this trial, the ORR was reached 53.8% in the group that received the combination treatment with 250 mg apatinib [65]. Of note, the DCR was 93.8%, and 6- and 9-month PFS reached 51.3 and 41.0%, respectively, which were significantly higher than those of the nivolumab-alone group [13, 65] (Table 1). This combination was evaluated as a first-line treatment in a phase III trial for advanced HCC (NCT03764293). Similarly, many other ICIs combined with angiogenesis inhibitors are being actively tested in phase III trials for HCC. For example, CS1003 (anti-PD-1 mAb) plus lenvatinib (NCT04194775) and durvalumab (anti-PD-L1 mAb) plus bevacizumab (NCT03847428) (other combination therapies are summarized in Table 2).

#### ICIs combined with other ICIs

The combination of ICIs with other ICIs has also achieved satisfactory outcomes in previous clinical trials [66]. In 2020, the ASCO published the results of a phase II trial involving durvalumab (anti-PD-L1 mAb) plus tremelimumab (anti-CTLA-4 mAb) treatment in a second-line setting for advanced HCC (NCT02519348). The high-dose combination treatment group (300 mg tremelimumab, T300) had an OS of 18.7 months, an ORR of 22.7%, and acceptable safety, while the other two monotherapy groups and the low-dose combination group (75 mg tremelimumab, T75) had poor survival and a limited response to the treatment [67]. T300 plus durvalumab is currently being evaluated as a first-line treatment for HCC in a phase III trial (NCT03298451). ICI combination treatments, including IBI310 (anti-CTLA-4 mAb) plus sintilimab injection (PD-1 inhibitor) are currently being evaluated in phase III trials (NCT04720716). Other combination therapies are summarized in Table 2.

#### Icaritin

Icaritin is a prenylflavonoid compound that is extracted from epimedium. Icaritin selectively modulates ERa36 and inhibits the initiation and growth of HCC through the IL-6/JAK2/STAT3 pathway [68]. More importantly, icaritin also plays an immunomodulatory role and inhibits PD-L1 expression [69, 70]. Given that basic studies have shown that icaritin has a promising anticancer effect, icaritin was tested in a phase I trial as an oral immune-modulating agent for HCC (NCT02496949). The results of this trial indicated that the median OS was 192 days, TTP was 141 days, and the toxicity was manageable [71]. Treatment benefit was observed in 46.7% of the patients, including a partial response rate of 6.7% and stable disease rate of 40% [71]. Among the patients who benefited from the treatment, the median OS was 488 days, preliminarily demonstrating the survival benefit of icaritin for patients with HCC [71]. Moreover, immune-inflammation dynamic biomarker analysis showed that reduced neutrophils and increased lymphocytes indicated better TTP, and a decrease in the ratio of platelets to lymphocytes was beneficial to OS [71]. Currently, icaritin is being evaluated for HCC in two phase III trials. (NCT03236649, NCT03236636).

### Agents in phase I/II trials

#### FGF19/FGFR4 signaling pathway

Fibroblast growth factor 19 (*FGF19*) and its receptor, fibroblast growth factor receptor 4 (*FGFR4*), are overexpressed in HCC and promote HCC progression by inhibiting apoptosis and promoting proliferation and invasion [72, 73]. In 2018, researchers improved the properties of BLU9931, the first small-molecule irreversible inhibitor that specifically targets FGFR4 in HCC, to design a novel, highly selective oral FGFR4 inhibitor, BLU554 (also known as fisogatinib) [74, 75]. Subsequently, a BLU554 was evaluated in a biomarker-based phase I trial for patients with FGF19 IHC+ advanced HCC (NCT02508467). The interim report of this trial showed a promising outcome with an ORR of 16% and DCR of 68% [75, 76]. Currently, BLU554 plus the anti-PD-L1 mAb CS1001 is being tested in a phase I/II trial for HCC (NCT04194801). Another FGFR4-selective inhibitor, H3B-6527, is also being evaluated in a phase I trial for HCC (NCT02834780). Compared with early FGFR4 inhibitors, H3B-6527 possesses a more powerful affinity for FGFR4, higher FGFR4 selectivity, and outstanding antitumor activity [75]. Furthermore, the cyclin-dependent kinase 4/6 (CDK4/6) inhibitor palbociclib facilitated tumor regression in combination with H3B-6527 in a xenograft model of HCC, which indicates a potential new strategy for HCC treatment [77].

#### PI3K/AKT/mTOR signaling pathway

The phosphoinositide 3-kinase (*PI3K*)/*AKT*/mammalian target of rapamycin (*mTOR*) pathway is the downstream signaling pathway of many receptor tyrosine kinases (*VEGFR*, *EGFR*, *PDGFR*, and *IGF-1R*) [78, 79]. It plays a dominant role in regulating cell growth and cancer survival [78].

SF1126, a new RGDS-conjugated LY294002 prodrug, exerts an antitumor role through dual PI3K/BRD4 inhibition and RAS/RAF/MAPK pathway blockade [80, 81]. In 2012, a phase I trial report indicated that SF1126 is well-tolerated by patients with advanced malignancies [82]. Subsequent preclinical research also confirmed the anti-HCC activity of SF1126 alone or in combination with sorafenib in vitro and in vivo, which provided key support for the evaluation of SF1126 in further trials for HCC [81]. Additionally, increasing evidence has shown that PI3K blockade facilitates the improvement of tumor susceptibility to immunotherapy [83, 84]. A phase I trial combining SF1126 with nivolumab for advanced HCC is actively underway (NCT03059147). Everolimus is a rapamycin analog that targets mTOR. However, everolimus failed to improve the OS of patients with HCC in the 2014 phase III EVOLVE-1 trial [85]. Recently, the pan-mTOR inhibitor sapanisertib (also known as MLN0128) was evaluated in an ongoing randomized phase I/II trial in patients with advanced or metastatic HCC (NCT02575339) [86]. Notably, a study shed light on the synergetic role of PD-1 inhibition and sapanisertib in suppressing HCC growth by eIF4E- and S6-mediated mechanisms [87]. Further clinical trials are needed to validate the combination efficacy of these agents for HCC.

#### TGF-β pathway

Transforming growth factor-β (TGF-β) is a tumor suppressor during the early phase of carcinogenesis and promotes cancer development during the late phase by inducing epithelial-to-mesenchymal transition and other mechanisms [88, 89]. Despite these two opposite roles, targeting the TGF-β pathway is still a promising strategy for cancer therapy. Galunisertib (LY2157299) is a small molecule inhibitor of TGF-β1 receptor type I. Studies have shown that galunisertib significantly disturbs HCC progression, and its combined use with sorafenib enhances the suppressive effect on HCC by overcoming sorafenib resistance [90, 91]. Based on these preclinical studies’ optimistic outcomes, a galunisertib was tested in a phase I/II trial as a monotherapy or in combination with sorafenib for HCC (NCT01246986). The trial results first demonstrated that galunisertib was well-tolerated, and low baseline AFP (AFP < 1.5 × ULN) and AFP or TGF-β responders (> 20% decrease from baseline) had better survival [92]. Subsequently, the results of galunisertib combined with sorafenib treatment showed that the TTP was 4.1 months, and the median OS reached 18.8 months in the 150 mg galunisertib combination cohort [93]. In the subgroup analysis, the TTP of the baseline AFP ≥ 400 ng/mL group or TGF-β ≥ 1956 pg/mL baseline median group was longer than that of the group below the median [93]. Furthermore, AFP responders had poorer OS than nonresponders (17.9 versus 20.6 months), while the OS of TGF-β responders was significantly longer than that of nonresponders (22.8 versus 12 months) [93]. Therefore, both baseline and response levels of AFP and TGF-β could be prognostic factors of survival. In addition, a preclinical study demonstrated that galunisertib enabled the regulation of T cell immunity and synergized with the antitumor effect of PD-1/L1 inhibitors [94]. Currently, galunisertib in combination with nivolumab for HCC is being evaluated in a phase I/II trial (NCT02423343).

#### Immune checkpoint bispecific antibodies (bsAbs)

Although CTLA-4, an immune checkpoint, has achieved long-lasting efficacy in tumors, severe immune-related toxicity limits its application [95]. At present, bsAbs are a promising and practical strategy to improve the therapeutic response and decrease immune-related AEs [96]. Recently, researchers designed a novel bsAb, AK104, that targets CTLA-4 and PD-1. The results of a phase I trial demonstrated that AK104 showed controllable safety and promising efficacy in advanced solid tumors, with an ORR of 28.6% and DCR of 47.6% in the group treated with doses ≥2 mg/kg (NCT03261011) [97]. Subsequently, AK104 combined with chemotherapy also presented an ORR of 57.9% and DCR of 94.7% in a phase I/II trial for advanced gastric and gastroesophageal junction cancer, which indicated the great potential of AK104 in combination with chemotherapy for cancer therapy [98]. Currently, the efficacy and safety of AK104 alone or in combination with lenvatinib for advanced HCC are being evaluated in two phase I/II trials (NCT04728321, NCT04444167). Another monovalent CTLA-4/PD-1 bsAb, MEDI5752, is also being actively tested in a phase I trial of advanced solid tumors (NCT03530397) [99]. Additionally, multiple innovative bsAbs for HCC treatment have been designed and developed in preclinical studies. For example, KN046 is a novel CTLA-4/PD-L1 bsAb, and is being tested in combination with TKIs for HCC in two phase I/II trials (NCT04542837, NCT04601610). Another bsAb targeting glypican 3 (GPC3) and CD47 can strengthen innate immune responses, which provides new insight and a potential new strategy for HCC therapy [100].

#### Adoptive cell transfer (ACT)

CAR-T cell therapy is an ACT approach involving the injection of T cells genetically engineered to express chimeric antigen receptors (CARs) that specifically recognize and target tumors [101, 102]. GPC3-specific CAR-T cells directly target GPC3, the effective epitope for HCC therapy, to exert an antitumor role [103, 104]. Presently, a relevant clinical trial is underway (NCT02905188). Notably, off-target toxicity and tumor heterogeneity are major concerns that present critical safety and efficacy issues. It was recently proposed that the disruption of PD-1 can enhance the persistence and infiltration of CAR-T cells into tumors and boost their anti-HCC effect, which provides a unique combination strategy for HCC treatment [105, 106]. IMA202, which utilizes the T cell receptor (TCR) gene extracted from tumor-reactive T cell clones to target tumor-specific antigens by genetically engineered internal normal T lymphocytes, was evaluated in another trial (NCT03441100) [107]. The different antigen recognition mechanisms between TCRs and CARs affect the types of target antigen and tumor evasion [108].

#### Oncolytic viruses

The principle of oncolytic virotherapy is the genetic modification of a virus to strengthen its specific cellular tropism, thereby allowing it to selectively replicate within cancer cells and induce tumor immunity, which results in tumor cell lysis and death without harming normal tissues [109, 110]. JX-594 (also known as Pexa-Vec) is an engineered vaccinia virus that selectively targets tumors by inactivating viral thymidine kinase (vTK) [111]. In previous clinical trials, JX-594 was shown to obtain good results in HCC treatment [111, 112]. However, the results of a subsequent randomized phase III trial, PHOCUS, in which JX-594 in combination with sorafenib was evaluated for HCC treatment, demonstrated that JX-594 did not improve treatment efficacy (NCT02562755) [113]. JX-594 combined with nivolumab is currently being tested in an ongoing phase I/II trial as a first-line treatment for advanced HCC (NCT03071094). Additionally, T-Vec (talimogene laherparepvec), a genetically engineered herpes simplex virus type I (HSV-1) [114], in combination with pembrolizumab treatment, is being tested in a phase I/II trial for HCC (NCT02509507).

## Emerging targets in preclinical trials

The exploration and excavation of novel targets is now a hot topic. In 2019, Tomas-Loba et al. [115] were surprised to find a new function of the p38γ protein in cell cycle regulation and hepatocarcinogenesis. This protein is highly similar in structure and mechanism to the CDK family, which is essential for controlling cell cycle progression [115]. Their research demonstrated that P38γ strongly inhibits the proliferation of HCC and acts as a potential therapeutic target for HCC [115]. NKG2A, expressed by NK cells and CD8+ T cells, serves as a novel immune checkpoint [116]. In 2018, van Montfoort et al. [117] showed that blocking NKG2A improved cancer vaccines’ clinical effect. Another study also revealed that the combination of anti-NKG2A and anti-PD-1/PD-L1 promoted antitumor activity by inducing CD8^+^ T cell memory [118]. Monalizumab is a humanized IgG4 antibody that specifically targets NKG2A and has been clinically evaluated in several cancers (NCT02921685, NCT02643550). TIGIT and CD96, which, together with the costimulatory receptor CD226, are akin to the CD28/CTLA-4 axis, have been shown that induce an antitumor immune response [119]. Furthermore, Schooten et al. [120] presented a unique cancer intracellular epitope—MAGE-A, which is expressed by multiple tumors of various histological origins and might be a meaningful immunotherapy target. Researchers also recently revealed that PCSK9, a critical negative regulator of low-density lipoprotein receptor (LDLR) that facilitates the degradation of LDLR, is associated with the regulation of T cell infiltration and CD8^+^ T cell immunosuppression in tumors [121, 122]. More importantly, PCSK9, an approved clinical treatment target for hypercholesterolemia with well-known toxicity profiles, significantly enhances the antitumor effect of anti-PD-1 antibodies [121, 122]. Thus, PCSK9 is another potential target for cancer immunotherapy.

## Agents for HCC treatment in China

In China, there were approximately 364,800 newly diagnosed cases of liver cancer and 318,800 liver cancer-related deaths in 2014; patients with liver cancer in China accounted for approximately 55% of the global liver cases [123]. Despite the fact that the incidence and mortality of HCC in China have decreased in recent years due to the popularization of infant vaccination and the control of viral infections, the number of patients with HCC remains substantial [123, 124]. As a result, research related to HCC treatment is of particular interest in China.

### Systemic treatment

In 2017, the Chinese Society of Clinical Oncology (CSCO) reported the Chinese subgroup’s outcome in the REFLECT trial of lenvatinib. The median OS was prolonged by 4.8 months for patients with HCC [17]. A rational explanation for this improvement is that lenvatinib has advantages in survival benefit for HBV-related HCC and HBV infection is the most frequent cause of hepatocarcinogenesis in China [1]. Furthermore, the secondary endpoints of the lenvatinib group were improved relative to the sorafenib group [17] (Table 1). Consequently, in 2018, lenvatinib was recommended as the first-line treatment for advanced HCC in China in the CSCO guidelines for hepatocellular carcinoma.

Substantial progress has been made in China in the development of ICIs and their application for the treatment of HCC. Toripalimab is an anti-PD-1 mAb developed by TopAlliance Biosciences Co., Ltd. In December 2018, toripalimab was approved by the National Medical Products Administration (NMPA) in China as a second-line treatment for unresectable or metastatic melanoma [125]. As the first anti-PD-1 mAb self-developed by China, toripalimab is of extraordinary significance. Subsequently, toripalimab was tested in various cancers and achieved positive outcomes in some [126–128]. Presently, several trials evaluating toripalimab as a treatment for HCC are underway (NCT03859128, NCT03867370). Tislelizumab (BGB-A317) is another anti-PD-1 mAb designed by BeiGene Co., Ltd. Tislelizumab is being evaluated in an ongoing phase III trial as a first-line treatment for patients with advanced HCC (NCT03412773). The main difference between tislelizumab and other general checkpoint inhibitors such as nivolumab is that other inhibitors possess the IgG4_s228p_ heavy chain, which maintains FcγR-binding function, whereas tislelizumab does not, and can be prevented from binding to macrophages (containing FcγR) that kill T cells and suppress the antitumor function [129]. The HengRui Medicine Co., Ltd. designed an anti-PD-1 mAb SHR-1210 (also known as camrelizumab). In 2019, they conducted a phase III trial to evaluate SHR-1210 plus apatinib as a first-line treatment for advanced HCC in China (CTR20182528). This trial was based on an encouraging outcome from a previously discussed phase I trial [65]. Subsequently, SHR-1210 was assessed in a phase II trial for advanced HCC performed at 13 study sites in China [130]. The results were favorable, with an ORR of 14.7%, a 6-month OS of 74.4%, and good antitumor activity in pretreated Chinese patients with advanced HCC, promoting the approval of SHR-1210 as a second-line treatment for advanced HCC in China [130]. The previously discussed CTLA-4/PD-1 bsAb AK104 was also independently researched and developed by Akeso, Inc. in China. Other agents being tested in clinical trials in China are summarized in Table 3.

**Table 3:** The ongoing clinical trials of hepatocellular carcinoma (HCC) in China

Agent	Targets	Conditions and Stage	Phase	Primary endpoint(s)	Biomarker	Companies	ID number^a^	Studystart
**Immunotherapy**								
Pembrolizumab	PD-1	Advanced; Second-line	III	OS	/	Merck Sharp & Dohme Corp.	CTR20160696	2017
Tislelizumab (BGB-A317)	PD-1	Unresectable; First-line	III	OS/Safety	/	BeiGene.	NCT03412773	2017
Toripalimab (JS001)	PD-1	Complete resection; Adjuvant	II/III	RFS	/	TopAlliance Biosciences Inc.	NCT03859128	2019
Camrelizumab (SHR-1210)	PD-1	Advanced; Second-line	II	ORR/OS (6 M)	/	Jiangsu HengRui Medicine Co., Ltd.	NCT02989922	2016
CAR-T cell	EpCAM	EpCAM positive	I/II	Toxicity	/	First Affiliated Hospital of Chengdu Medical College.	NCT03013712	2017
Infusion of iNKT cells and CD8^+^T cells	Lysis tumor cells	Advanced	I/II	AEs/ORR	/	Shanghai Public Health Clinical Center.	NCT03093688	2017
KN035	PD-L1	Advanced	I	DLTs/AEs/ORR	/	3D Medicines (Sichuan) Co., Ltd.; Alphamab Co., Ltd.	NCT03101488	2017
GLS-010 injection	PD-1	Advanced	I	AEs/AA	PD-L1	Harbin Gloria Pharmaceutical Co., Ltd.	CTR20170692	2017
iNKT cells	Lysis tumor cells	Relapsed, advanced	I	AEs	/	Beijing YouAn Hospital.	NCT03175679	2017
ET1402L1-CAR-T cells	AFP	AFP+, advanced	I	DLT/Toxicity	AFP	Aeon Therapeutics (Shanghai) Co., Ltd.	NCT03349255	2017
GPC3-T2-CAR-T cells	GPC3	GPC3+	I	DLT	/	Second Affiliated Hospital of Guangzhou Medical University.	NCT03198546	2017
**Anti-angiogenesis**								
Donafenib	VEGFR, PDGFR, RAF	Advanced; First-line	III	OS	/	Suzhou Zelgen Biopharmaceuticals Co., Ltd.	NCT02645981	2016
Lenvatinib (E7080)	VEGFR1–3, FGFR1–4, PDGFR α, RET, and KIT	Unresectable; First-line	III	OS	/	Eisai Co., Ltd.	CTR20131648	2014
Muparfostat (PI-88)	FGF1–2, VEGF	Resected; Adjuvant	III	DFS	/	Medigen Biotechnology Corp.	CTR20131019	2015
Brivanib (ZL-2301)	VEGFR, FGFR	Advanced; Second-line	II	DCR (3 M)	/	Zai Lab Pharmaceutical (Shanghai) Co., Ltd.	CTR20170216	2017
Lucitanib (AL3810)	FGFR1–2, VEGFR1–3	Advanced or metastatic	Ib	AE	/	Shanghai Institute of Materia Medica; Academia Sinica.	CTR20160271	2016
Metatinib Trometamol tablets	MET/VEGFR2	Advanced or metastatic	Ib	AE	MET	Simcere Pharmaceutical Co., Ltd.	CTR20150743	2015
Chiauranib	VEGFR, PDGFRa, KIT, Aurora B and CSF-1R	Advanced	I	PFR (16 W)	/	Chipscreen Biosciences, Ltd.	NCT03245190	2018
**Cell cycle inhibitors and Anti-proliferation**								
Erdafitinib (JNJ-42756493)	FGFR	Advanced; FGF19 amplification	I/II	RP2D/ORR	/	Janssen Research & Development, LLC.	NCT02421185	2015
ATG-008 (CC-223)	mTORC1/2	HBV+, Advanced; Second-line	II	PK/AEs/ORR	TORC1/2 and others	Antengene Corporation.	NCT03591965	2018
**Combination therapy**								
PD-1 Antibody Plus Lenvatinib	PD-1 + VEGFR1–3, FGFR1–4, PDGFR α, RET, and KIT	Advanced	III	OS	/	Sun Yat-sen University.	NCT03744247	2019
HLX10 plus HLX04	PD-1+ VEGF	Locally Advanced or Metastatic; First-line	III	OS/PFS	/	Shanghai Henlius Biotech.	NCT04465734	2020
Durvalumab plus Tremelimumab	PD-L1 + CTLA-4	Unresectable; First-line	III	OS/AE	/	MedImmune LLC.	CTR20180607	2018
SHR-1210 plus FOLFOX4	PD-1 + DNA Synthesis	Advanced; First-line	III	OS	/	Jiangsu HengRui Medicine Co., Ltd.	NCT03605706	2018
SHR-1210 plus Apatinib	PD-1 + VEGFR-2	Advanced; First-line	III	OS/PFS	/	Shanghai HengRui Medicine Co., Ltd.	CTR20182528	2019
Sintilimab plus IBI305	PD-1 + VEGF	Advanced; First-line	II/III	OS/ORR	/	Innovent Biologics (Suzhou) Co. Ltd.	NCT03794440	2019
AK105 plus Anlotinib or AK105 plus Bevacizumab	PD-1 + VEGFR, PDGFR, FGFR, KIT or PD-1 + VEGFA	Unresectable; First-line	Ib/II	ORR	/	Akeso (Guangdong) Biopharma, Inc.	CTR20182026	2018
PD-1 mAb plus PolyIC	PD-1 + TLR3	Unresectable	II	ORR	AFP	Second Affiliated Hospital, School of Medicine, Zhejiang University.	NCT03732547	2018
PHY906 plus Sorafenib	Chinese herbal formulation+VEGFRs, KIT, PDGFRs, and RAF	Advanced	I	RP2D	/	City of Hope Medical Center.	NCT01666756	2013
**Traditional Chinese medicine**								
Xihuang Capsules	/	Resected	IV	recurrence rate	/	Shuqun Cheng, Eastern Hepatobiliary Surgery Hospital.	NCT02399033	2015
Icaritin	ERa36	Advanced; HBV-Related	III	OS	PD-L1, hnRNPAB1, IL-6 and others	Beijing Shenogen Biomedical Co., Ltd.	NCT03236636	2017
Icaritin	ERa36	PD-L1+, advanced	III	OS	PD-L1, hnRNPAB1, IL-6 and others	Beijing Shenogen Biomedical Co., Ltd.	NCT03236649	2017
Ursolic acid nanoliposomes injection	IKKβ	Advanced;	I	ORR	/	Wuhan Liyuanheng Pharmaceutical Co., Ltd.	CTR20140395	2016
Chlorogenic acid	cell cycle	Advanced;	I	DLT/MTD	/	Sichuan Jiuzhang Biotech Co., Ltd.	CTR20130586	2014
Jiu-wei-zhen-xiao Granule	/	Advanced; Unresectable	I	PFS	/	Zhong Wang, China Academy of Chinese Medical Sciences.	NCT03851471	2019
**Other mechanisms**								
K-001 (Marine biological products)	anti-inflammation, anti-angiogenesis	Advanced	III	OS	AFP	Beijing Huashi Tianfu Biomedical Technology Co., Ltd.	CTR20132910	2014
IFN-alpha	pro-apoptosis, anti-proliferation	Resected; low miR-26 expression	III	DFS	miR-26	Fudan University.	NCT01681446	2012
Galunisertib (LY2157299)	TGF-β	Advanced; First-line	II	OS	/	Eli Lilly and Company.	CTR20150343	2015
Boanmycin Hydrochloride for Injection	cytotoxic agent	/	II	ORR	/	DIKANG Pharmaceutical.	CTR20140308	2015
PT-112	pro-apoptosis, immunogenic cell death inducer	Advanced	I/II	AEs/DLTs/DCR	/	SciClone Pharmaceuticals.	NCT03439761	2018
Hemay102	cytotoxic agent	Advanced;	I	AE	/	Hainan Hailing Chemipharma Corporation Limited.	CTR20181497	2018
ZSP1241	/	Advanced	I	MTD/DLT/AEs	/	Guangdong Zhongsheng Pharmaceutical Co., Ltd.	NCT03734926	2018
CGX1321	WNT	Advanced	I	AEs	/	Curegenix Inc.	NCT03507998	2017

*Abbreviations*: *PD-1* programmed cell death-1, *PD-L1* programmed cell death ligand 1, *EpCAM* epithelial cell adhesion molecule, *AFP* alpha fetoprotein, *GPC3* glypican 3, *VEGFR* vascular endothelial growth factor receptor, *VEGF* vascular endothelial growth factor, *PDGFR* platelet derived growth factor receptor, *FGFR* fibroblast growth factor receptor, *FGF* fibroblast growth factor, *PDGFR* platelet derived growth factor receptor, *CSF-1R* colony stimulating factor 1 receptor, *mTORC1/2* mechanistic target of rapamycin kinase complex 1 and 2, *TLR3* toll like receptor 3, *CTLA-4* cytotoxic T lymphocyte–associated antigen-4, *IKKβ* inhibitor of nuclear factor kappa B kinase subunit beta, *ERa36* estrogen receptor-alpha 36, *TGF-β* transforming growth factor beta, *OS* overall survival, *RFS* recurrence free survival, *ORR* objective response rate, *DLT* dose limited toxicity, *AEs* adverse events, *DFS* disease free survival, *DCR* disease control rate, *PFR* progression free rate, *RP2D* recommended phase II dose, *PK* pharmacokinetic, *MTD* maximum tolerated dose, *PFS* progress free survival

^a^ID number: IDs starting with NCT are from clinicaltrials.gov, while IDs starting with CTR are from chinadrugtrials.org.cn/index.html

### Traditional Chinese medicine

The TCM has a long history and has been widely applied for the treatment of various diseases. A variety of TCMs have been used for HCC treatment in China for many years with certain efficacy and good safety, including elemene, cinobufotalin, and kanglaite injection, despite the lack of high-quality and multicenter clinical trials [131]. PHY906 is a Chinese herbal formula that was developed from the Huang Qin Tang herbal mixture [132]. It consists of the following four herbal ingredients: Glycyrrhiza uralensis Fisch, *Paeonia lactiflora* Pall, Scutellaria baicalensis Georgi, and *Ziziphus jujuba* Mill [132]. The results of previous early-phase trials showed promising efficacy of PHY906 combined with capecitabine for HCC [133, 134]. The results of preclinical studies also indicated that PHY906 enhanced the anti-HCC efficacy of sorafenib by promoting tumor apoptosis and autophagy and regulating inflammation in the tumor microenvironment (TME) [132]. PHY906 is being evaluated in a phase II trial in combination with sorafenib for HCC (NCT04000737). Other TCMs under clinical trials are summarized in Table 3.

Due to the integration of TCM experience with modern medical theory systems, the long-term puzzles of weak absorption, low bioavailability, and apparent side effects of TCMs have been resolved gradually, for example, the development of the novel micellar system GA-PEG-SS-PLGA [135]. Furthermore, future studies need to identify the active substances in TCM compounds and explore the specific functional mechanisms of the anticancer effect of these substances. Of note, evidence-based medicine requires not only mechanistic knowledge but also clinical research. Jin-Ling Tang proposed an efficacy-driven strategy for TCM research, emphasizing that drug mechanism research can be meaningful only if it is clinically efficacious [136]. Therefore, it is also essential to promote clinical trials of traditional medicine in the future.

## Challenges in HCC treatment

The efficacy of sorafenib fails to meet the expectations of better survival benefit, and although immunotherapy has been advantageous for HCC treatment, a durable and effective response only occurs in a small proportion of patients with HCC [137]. All of these facts indicate that challenges in HCC treatment still exist.

### Therapeutic resistance

The survival of patients with advanced HCC treated with sorafenib is limited to 1 year [138]. This limitation may partially be attributed to drug resistance, and its potential mechanisms include the activation of *EGFR*, epithelial-mesenchymal transition (EMT), cancer stem cell (CSC) presence, or tumor-initiating cell (TIC) involvement [139, 140]. Furthermore, the TME plays a substantial role in the development of tumor resistance to therapeutics [141]. A variety of cells in addition to tumor cells make up the TME, which plays a suppressive role in the antitumor effect by multiple mechanisms, such as restriction of T cell accumulation in tumors and T cell exhaustion or dysfunction [141, 142]. It is urgent to develop strategies to overcome drug resistance. First, resistance-related molecules have been targeted. Cetuximab (EGFR-targeted mAb) is a preferred candidate. Despite negative outcomes in several trials, cetuximab’s powerful potential is still worth exploring in the future [143]. Second, combinations of multiple agents that target different pathways have been evaluated. For example, the results of one study demonstrated that anti-CXCR4 in combination with anti-PD-1 and sorafenib is beneficial for patients with HCC [144]. Finally, predictive biomarker-based population selection is an important future topic of investigation. Genetic heterogeneity, which partially explains drug resistance, determines the significance of personalized therapy [140]. Hence, exploring predictive biomarkers facilitates the implementation of precise treatment.

### Biomarkers

Although the biomarkers’ pivotal role in therapy response has been emphasized for several years, clinically valuable biomarkers remain lacking. In 2016, Zhu et al. [145] developed a large-scale biomarker-related study under the phase III SEARCH trial [146]. They revealed that the upregulation of HGF, VEGFA, and VEGFC expression and the downregulation of plasma KIT levels were closely related to poor clinical outcomes in patients with HCC treated with sorafenib with or without erlotinib [145]. Regrettably, there has been no more in-depth exploration to distinguish the clinical benefit differences between sorafenib alone or in combination with erlotinib. Additionally, many molecules, such as tumor-associated macrophages (TAMs), PD-L1, tumor-infiltrating lymphocytes (TILs), and a strategy referred to as the immunoscore, have been described as potential biomarkers for immunotherapy in cancers [147–149]. However, not all of these candidates are equally appropriate for HCC, such as PD-L1 [13]. The efficacy and clinical value of these hypothetical biomarkers remain to be verified in future trials.

### Treatment safety

With the promising efficacy of immunotherapy unveiled, more clinical trials are needed to assess ICIs in various tumors. As a result, severe immune-related AEs have been discovered, in particular cardiotoxicity. In 2018, Moslehi et al. [150] analyzed 101 cases of fatal myocarditis after ICI treatment. They emphasized the high fatality risk of this AE, especially with the combination of PD-1 and CTLA-4 mAbs (death rate of 36% with monotherapy and 67% with the combination treatment) [150]. Previous studies also reported fulminant myocarditis in individual patients [151, 152]. In addition, as normal tissues also express partial tumor antigens, adoptive cell treatment might harm healthy tissues by targeting these tumor antigens (also known as autoimmune toxicity) [153]. Promisingly, several tactics to resolve this problem have been proposed, including TNF-α- or IL-6-targeted drugs and engineered T cells expressing inducible suicide genes [153–156]. Additionally, engineered T cells expressing two kinds of CARs or TCRs are promising strategies that target two tumor antigens simultaneously and while sparing normal tissues that do not express the two malignancy-related antigens together [153–155].

## Conclusions

Looking back on the history of HCC drug development, many promising drugs have failed in phase III clinical trials (summarized in Supplementary Table S1). Owing to the continuous innovation of scientific research, abundant studies related to targeted therapy of HCC have been exceedingly rewarding in recent years. Specifically, the FDA approved several antiangiogenic agents and immune checkpoint inhibitors for HCC between 2017 and 2020, and multiple agents are being evaluated in current clinical trials. However, the moderate efficacy of targeted therapy, the lack of biomarkers for therapeutic responsiveness, and the limitations in achieving a durable response to immune inhibitors still exist. In the past decade, the development of sequencing technologies has boomed, in particular that of high-throughput sequencing, which has enabled rapid large-scale genome sequencing at a lower cost [157]. However, the deficiency in accuracy limits the ability of high-throughput sequencing to delineate a comprehensive molecular map; for example, it is challenging to detect rare genetic variations with this sequencing approach [158]. Precision medicine represents one of the goals of tailoring healthcare for specific patients based on their data from various technologies, in particular genetic sequencing [159]. Consequently, there will be more stringent requirements for sequencing in terms of efficiency, accuracy, stability, and low price in the future. In addition, proteomics is being advanced and perfected gradually, but the implementation of high-sensitivity and high-accuracy proteomics technology is still a challenge. Consequently, the following possible directions should be considered. (i) Elucidation of the molecular mechanisms of HCC from multiple perspectives, such as genetics and epigenetics, cell-cell interactions, and the roles of the TME. (ii) Construction of stratified treatments based on predictive biomarkers to enable individualized treatment. Josep M. Llovet and Virginia Hernandez-Gea discussed two trial designs related to this concept in their review [138], namely proof-of-concept trials and biomarker-based enrichment trials. They emphasized that we should not only concentrate on multitarget drugs that aimed toward eligible patients but also specific targeted drugs that target a subpopulation of patients characterized by molecular aberrations. (iii) Exploration of extracts from traditional Chinese medicines, such as artemisinin and chloroquine derivatives [160, 161]; the large number of patients with HCC in China presents an opportunity to gain more evidence of the efficacy of these drugs and experience in their application. In summary, with the increasing focus on HCC research and the rapid development of molecular biotechnology, more mysteries of HCC will be resolved comprehensively and deeply, and more treatment strategies will be presented.

## Supplementary Information

**Additional file 1: Table S1.** The agents that failed in phase III clinical trials for HCC

## Data Availability

The datasets generated and analyzed during the current study are available in the US National Library of Medicine repository (https://clinicaltrials.gov) and the China drug trials repository (http://www.chinadrugtrials.org.cn/index.html).

## Abbreviations

HCC: Hepatocellular carcinoma
TCM: Traditional Chinese medicine
FDA: United States Food and Drug Administration
PD-1: Programmed cell death-1
PD-L1: Programmed cell death ligand-1
CTLA-4: Cytotoxic T lymphocyte–associated antigen-4
ICB: Immune checkpoint blockade
ICIs: Immune checkpoint inhibitors
OS: Overall survival
ORR: Objective response rate
DCR: Disease control rate
ESMO: European Society for Medical Oncology
AEs: Adverse events
mAb: Monoclonal antibody
PRR: Partial response rate
ACT: Adoptive cell transfer
CARs: Chimeric antigen receptors
TCRs: T cell receptors
GPC3: Glypican 3
vTK: Viral thymidine kinase
HSV-1: Herpes simplex virus type I
HFSR: Hand-foot skin reaction
PFS: Progression-free survival
TTP: Time to progression
HAIC: Hepatic arterial infusion chemotherapy
TKI: Tyrosine kinase inhibitor
AFP: α-fetoprotein
ASCO: American Society of Clinical Oncology
FGF19: Fibroblast growth factor 19
FGFR4: Fibroblast growth factor receptor 4
PI3K: Phosphoinositide 3-kinase
mTOR: Mammalian target of rapamycin
TGF-β: Transforming growth factor-β
bsAbs: Bispecific antibodies
CDK: Cyclin-dependent kinase
LDLR: Low-density lipoprotein receptor
EMT: Epithelial-mesenchymal transition
CSCs: Cancer stem cells
TICs: Tumor-initiating cells
TME: Tumor microenvironment
TAMs: Tumor-associated macrophages
TILs: Tumor-infiltrating lymphocytes
CSCO: Chinese Society of Clinical Oncology
NMPA: National Medical Products Administration
