# Supplementary material for: Advances in drug development for hepatocellular carcinoma: clinical trials and potential therapeutic targets
Source: J Exp Clin Cancer Res. 2021 May 18;40:172. doi: 10.1186/s13046-021-01968-w (PMC8130401; doi:10.1186/s13046-021-01968-w)
Supplement: Supplementary file 1 — Additional file 1: Table S1. The agents that failed in phase III clinical trials for HCC [file 13046_2021_1968_MOESM1_ESM.docx]

**Table S1.** Agents that failed in phase III clinical trials for HCC.

| **Agent** | **Targets** | **Study design** | **Sample size** | **OS** | **Other results** |
| --- | --- | --- | --- | --- | --- |
| Brivanib vs Sorafenib (BRISK-FL) [1] | FGF and VEGF | First-line  Randomized Multinational  Double-blind | 1,155 patients (Brivanib=577; Sorafenib=578) | Brivanib: 9.5 months Sorafenib: 9.9 months HR: 1.06 (95% CI, 0.93 to 1.22, P=0.3730) | **TTP:** Brivanib: 4.2 months Sorafenib: 4.1 months HR: 1.01 (95% CI, 0.88 to 1.16; P=0.8532) **ORR**: 12% **DCR**:66% |
| Brivanib vs Placebo (BRISK-PS) [2] | FGF and VEGF | Second-line Randomized Multicenter  Double-blind | 395 patients (Brivanib=263; Placebo=132) | Brivanib: 9.4 months Placebo: 8.2 months HR: 0.89 (95.8% CI, 0.69 to 1.15, P=0.3307) | **TTP:** Brivanib: 4.2 months Placebo: 2.7 months HR: 0.56 (95% CI, 0.42 to 0.76; P< 0.001) **ORR**: 10% **DCR**: 61% |
| Sunitinib vs Sorafenib [3] | VEGFR | First-line  Randomized | 1074 patients (Sunitinib=530; Sorafenib=544) | Sunitinib: 7.9 months Sorafenib: 10.2 months HR: 1.30 (95% CI, 1.13 to 1.50, P=0.9990) | **TTP:** Sunitinib: 4.1 months Sorafenib: 3.8 months HR: 1.13 (95% CI,0.98 to 1.31; P =0.8312) **PFS**: Sunitinib: 3.6 months Sorafenib: 3.0 months HR: 1.13 (95% CI, 0.99 to 1.30; P=0.8785) |
| Sorafenib Plus Erlotinib vs Sorafenib [4] | EGFR | First-line  Randomized Multinational | 720 patients (Sorafenib+Erlotinib=362; Sorafenib+Placebo=358) | Sorafenib+Erlotinib: 9.5 months Sorafenib+Placebo: 8.5 months HR: 0.929; (95% CI, 0.781 to 1.106, P=0.408) | **TTP:** Sorafenib+Erlotinib: 3.2 months  Sorafenib+Placebo: 4.0 months HR: 1.135 (95% CI, 0.944 to 1.366 P=0.18) **ORR**: 6.6% **DCR:** 43.9% |
| Linifanib vs Sorafenib [5] | VEGFR and PDGFR | Frist-line  Randomized  Open-label Multicenter | 1,035 patients  （Linifanib=514;Sorafenib=521) | Linifanib: 9.1 months Sorafenib: 9.8 months HR: 1.046 (95% CI, 0.896 to 1.221) | **TTP:** Linifanib: 5.4 months Sorafenib: 4.0 months HR: 0.759 (95% CI, 0.643 to 0.895; P<0.001) **ORR**: 13% |
| Everolimus vs Placebo [6] | mTOR | Second-line Randomized International  Double-blind | 546 patients  （Everolimus=362;Placebo=184) | Everolimus: 7.6 months Placebo: 7.3 months HR: 1.05 (95% CI, 0.86 to 1.27, P=0.68) | **TTP**: Everolimus: 3.0 months Placebo: 2.6 months HR: 0.93 (95% CI, 0.75-1.15)  **DCR**: 56.1% |
| Tivantinib vs placebo (METIV-HCC) [7] | Met | Second-line Randomized Multicenter  Double-blind | 340 patients (Tivantinib=226；placebo=114) | Tivantinib: 8.4 months Placebo: 9.1 months HR: 0.97; (95% CI 0.75 to 1.25; p=0.81). | **TTP**: Tivantinib: 2.4 months Placebo: 3.0 months HR: 0.96 (95% CI, 0.74 to 1.25; p=0.76) **PFS**: Tivantinib: 2.1 months Placebo: 2.0 months HR: 0.96 (95% CI, 0.75 to 1.22; p=0.72) **DCR**: 50% |

Abbreviations

FGF, fibroblast growth factor; VEGF, vascular endothelial growth factor; EGFR, epidermal growth factor receptor; PDGFR, platelet derived growth factor receptor; mTOR, mechanistic target of rapamycin; OS, overall survival; HR, hazard ratio; CI, confidence interval; TTP, time to progress; ORR, objective response rate; DCR, disease control rate; PFS, progress free survival;

1. Johnson PJ, Qin S, Park JW et al. Brivanib versus sorafenib as first-line therapy in patients with unresectable, advanced hepatocellular carcinoma: results from the randomized phase III BRISK-FL study. J Clin Oncol 2013; 31: 3517-3524.

2. Llovet JM, Decaens T, Raoul JL et al. Brivanib in patients with advanced hepatocellular carcinoma who were intolerant to sorafenib or for whom sorafenib failed: results from the randomized phase III BRISK-PS study. J Clin Oncol 2013; 31: 3509-3516.

3. Cheng AL, Kang YK, Lin DY et al. Sunitinib versus sorafenib in advanced hepatocellular cancer: results of a randomized phase III trial. J Clin Oncol 2013; 31: 4067-4075.

4. Zhu AX, Rosmorduc O, Evans TR et al. SEARCH: a phase III, randomized, double-blind, placebo-controlled trial of sorafenib plus erlotinib in patients with advanced hepatocellular carcinoma. J Clin Oncol 2015; 33: 559-566.

5. Cainap C, Qin S, Huang WT et al. Linifanib versus Sorafenib in patients with advanced hepatocellular carcinoma: results of a randomized phase III trial. J Clin Oncol 2015; 33: 172-179.

6. Zhu AX, Kudo M, Assenat E et al. Effect of everolimus on survival in advanced hepatocellular carcinoma after failure of sorafenib: the EVOLVE-1 randomized clinical trial. JAMA 2014; 312: 57-67.

7. Rimassa L, Assenat E, Peck-Radosavljevic M et al. Tivantinib for second-line treatment of MET-high, advanced hepatocellular carcinoma (METIV-HCC): a final analysis of a phase 3, randomised, placebo-controlled study. Lancet Oncol 2018; 19: 682-693.
